# Automated Messaging Program to Facilitate Systematic Home Blood Pressure Monitoring: Qualitative Analysis of Provider Interviews

**DOI:** 10.2196/51316

**Published:** 2023-12-04

**Authors:** Julian Einhorn, Andrew R Murphy, Shari S Rogal, Brian Suffoletto, Taya Irizarry, Bruce L Rollman, Daniel E Forman, Matthew F Muldoon

**Affiliations:** 1 Syneos Health Bethesda, MD United States; 2 Division of Cardiology Department of Medicine Emory University School of Medicine Atlanta, GA United States; 3 Dissemination and Implementation Science Collaboration Department of Medicine University of Pittsburgh School of Medicine Pittsburgh, PA United States; 4 Division of Gastroenterology Department of Medicine University of Pittsburgh School of Medicine Pittsburgh, PA United States; 5 Department of Emergency Medicine Stanford University School of Medicine Palo Alto, CA United States; 6 Department of Advanced Analytics and Population Health Highmark Health Enterprise Pittsburgh, PA United States; 7 Center for Behavioral Health, Media and Techology Department of Medicine University of Pittsburgh School of Medicine Pittsburgh, PA United States; 8 Division of General Internal Medicine Department of Medicine University of Pittsburgh School of Medicine Pittsburgh, PA United States; 9 Division of Geriatrics Department of Medicine University of Pittsburgh School of Medicine Pittsburgh, PA United States; 10 Geriatrics, Reseach, Education and Clinical Care (GRECC) VA Pittsburgh Healthcare System Pittsburgh, PA United States; 11 Division of Cardiology Department of Medicine University of Pittsburgh School of Medicine Pittsburgh, PA United States; 12 UPMC Hypertension Center Heart and Vascular Institute UPMC Healthcare System Pittsburgh, PA United States

**Keywords:** mHealth, digital intervention, qualitative research, provider stakeholders, hypertension, home blood pressure monitoring, implementation research, short-messaging system, remote monitoring, qualitative analysis, messaging program, blood pressure, monitoring, cardiovascular, disease, text messaging, text mining, self-management, mobile phone

## Abstract

**Background:**

Hypertension is a leading cause of cardiovascular and kidney disease in the United States, yet blood pressure (BP) control at a population level is poor and worsening. Systematic home BP monitoring (HBPM) programs can lower BP, but programs supporting HBPM are not routinely used. The MyBP program deploys automated bidirectional text messaging for HBPM and disease self-management support.

**Objective:**

We aim to produce a qualitative analysis of input from providers and staff regarding implementation of an innovative HBPM program in primary care practices.

**Methods:**

Semistructured interviews (average length 31 minutes) were conducted with physicians (n=11), nurses, and medical assistants (n=6) from primary care settings. The interview assessed multiple constructs in the Consolidated Framework for Implementation Research domains of intervention characteristics, outer setting, inner setting, and characteristics of individuals. Interviews were transcribed verbatim and analyzed using inductive coding to organize meaningful excerpts and identify salient themes, followed by mapping to the updated Consolidated Framework for Implementation Research constructs.

**Results:**

Health care providers reported that MyBP has good ease of use and was likely to engage patients in managing their high BP. They also felt that it would directly support systematic BP monitoring and habit formation in the convenience of the patient’s home. This could increase health literacy and generate concrete feedback to raise the day-to-day salience of BP control. Providers expressed concern that the cost of BP devices remains an encumbrance. Some patients were felt to have overriding social or emotional barriers, or lack the needed technical skills to interact with the program, use good measurement technique, and input readings accurately. With respect to effects on their medical practice, providers felt MyBP would improve the accuracy and frequency of HBPM data, and thereby improve diagnosis and treatment management. The program may positively affect the patient-provider relationship by increasing rapport and bidirectional accountability. Providers appreciated receiving aggregated HBPM data to increase their own efficiency but also expressed concern about timely routing of incoming HBPM reports, lack of true integration with the electronic health record, and the need for a dedicated and trained staff member.

**Conclusions:**

In this qualitative analysis, health care providers perceived strong relative advantages of using MyBP to support patients. The identified barriers suggest the need for corrective implementation strategies to support providers in adopting the program into routine primary care practice, such as integration into the workflow and provider education.

**Trial Registration:**

ClinicalTrials.gov NCT03650166; https://tinyurl.com/bduwn6r4

## Introduction

Hypertension is the leading cause of morbidity worldwide [1]. It affects 100 million US adults, most of whom have uncontrolled hypertension [2]. Many factors contribute to uncontrolled hypertension but particularly vexing are patient nonadherence to prescribed medications and lifestyle modifications, and provider reluctance to advance pharmacotherapy, termed *therapeutic inertia* [3,4]. Therapeutic inertia is exacerbated by uncertainty regarding data accuracy, as patients either do not record any blood pressure (BP) data at home or fail to organize readings in a manner that is verifiable and serviceable by providers [4-6]. Providers recognize several barriers to home BP monitoring (HBPM), some of which might be addressed using new technologies [7].

Interventions to facilitate collection and reporting of HBPM more reliably and systemically may combat therapeutic inertia by improving provider confidence in submitted HBPM readings. Some programs include ancillary self-management supports such as BP measurement reminders, automated feedback, and educational resources [8-10]. Critically, systematic reviews of randomized clinical trials have shown that HBPM coupled with a supporting resource lowers BP [11]. Due to its promise and advantages, US guidelines now broadly recommend HBPM in the diagnosis and management of hypertension [12,13]. However, implementation has not been systematized and practicality is uncertain. Some tested programs have employed pharmacists to manage pharmacotherapy, while others used case managers who followed up on readings by calling patients and offering verbal education [11]. These resource-intensive interventions have limited scalability and lack clear benefit to cost ratios [10,11]. Programs that are automated may have superior utility provided they are effective in engaging patients in systematic HBPM and supporting disease self-management. Still, designers must consider barriers to engagement with technologically based HBPM programs, particularly among older, rural, and other disadvantaged communities.

Automated communication programs using mobile phones to link patients and their providers have shown promise [14-18], including among underserved populations [19-22]. Previous work has highlighted that awareness of BP status and goals can enhance adherence to lifestyle recommendations and medication [23,24]. Widespread cell phone ownership with unlimited texting (SMS text messaging) makes SMS text messaging an attractive conduit through which automatic programs can operate without reliance on broadband, local Wi-Fi or uploaded apps [25]. SMS text messaging–based programs also have other benefits such as being simple and proactive that allow for high user engagement.

The goal of our program, MyBP, is to support both patient and provider in the management of hypertension through the above measures and ultimately control BP. The core feature is an automated SMS text messaging program systematically collecting longitudinal HBPM data and providing tailored BP feedback. MyBP also includes educational videos and provider reports summarizing BP trends [8]. MyBP has been developed in 3 phases spanning focus groups and feasibility studies using varied clinical settings [8]. Throughout this process, the developers of MyBP have received both formal and informal stakeholder feedback. In a qualitative analysis of patient interviews, participants reported improved understanding of, and motivation surrounding, healthy behaviors [26]. Objective engagement with MyBP over a period of 25 weeks further supports usefulness and implementation feasibility [27].

The goal of the current study was to collect and analyze providers’ perception of the barriers to and facilitators of MyBP implementation in primary care. Using semistructured interviews, we sought to identify unmet needs by current HBPM practices, areas of refinement within the MyBP program, and strategies for implementation. Artificial intelligence was not used in any part of the research or in writing this paper.

## Methods

### Study Design

In a qualitative analysis of stakeholder input from primary care providers and staff, this study assesses strengths and weaknesses of the current MyBP program, particularly the facilitators and barriers to implementation in primary care practices. Textbox 1 provides a summary of the MyBP program. Our methods relied on an implementation-focused formative evaluation using the Stetler et al [28] typology. The goals were primarily to identify actionable barriers to implementation while also identifying facilitating factors for implementation that could potentially be augmented. A convenience sample of primary care physicians, nurses, and medical assistants from local practices were recruited. Participants received an information packet providing a general overview of all the functions of the MyBP program and an example of the provider BP report. Some providers had had personal experience with MyBP while others had knowledge of the program only through printed materials and oral description. Interviews were conducted either in-person or via telephone by one of the authors (JE or TI) between January 2018 and September 2019.

**MyBP program overview.**

MyBP is a patient-facing, automated, bidirectional SMS text messaging program providing support of hypertension self-monitoring and self-management. It also generates blood pressure summary reports for providers. The program is delivered to patients via any text-capable phone and requires no internet connection or special equipment. Patient-submitted blood pressure readings are sent via SMS text messaging and collected in a secure server for processing.

- Upon enrollment, participants were given access and instructed to watch several health-education videos on hypertension by Emmi Solutions, Inc.
- Once enrolled, MyBP sent text messages supporting personalized and scheduled morning and evening blood pressure self-measurement.
- Each submitted prescheduled reading prompted confirmation and personalized blood pressure feedback.
- Patients received periodic tips to promote better health behaviors and were provided with continued access to educational videos.
- General guidance is offered when extremely low or high blood pressure readings are submitted.
- Monthly blood pressure reports were faxed automatically to primary care provider offices.

### Interview Development

The Consolidated Framework for Implementation Research (CFIR) was used to formulate interview questions [29]. This practical and theory-based guide for systematically assessing potential barriers and facilitators is used to either tailor implementation strategies and adaptations for the innovation being implemented or to explain outcomes of implementations. The CFIR includes 5 domains: characteristics of the intervention, the characteristics of individuals, implementation process, and the inner and outer settings or contexts. Each domain contains multiple constructs. It is not practical to cover every construct in an interview, so a group deliberation strategy was used between authors MFM and SSR to determine the most relevant constructs to address given the innovation under study in the context of primary care office practice. The selected CFIR constructs are listed in Multimedia Appendix 1. These CFIR constructs and definitions were then used to compose the interview questions and additional probes to elucidate details depending on the initial responses received. The interview text is found in Multimedia Appendix 1.

### Coding and Analysis

All interviews were recorded and transcribed verbatim by the investigators. Transcripts were thematically analyzed using an inductive coding approach [30,31]). This was accomplished through multiple in-depth readings of the transcripts by investigators (JE and ARM) to find meaningful excerpts related to the implementation of MyBP. These excerpts were then coded and grouped into categories based upon similar semantic or explicit content. The same investigators reviewed the transcripts and coded material independently. The unique coded excerpts were then discussed in detail to reach a consensus and add or subtract from the agreed-upon list of coded segments of text. If a new code was developed, all previously reviewed interviews were reanalyzed to determine the presence or absence of the new code.

**Figure 1:**
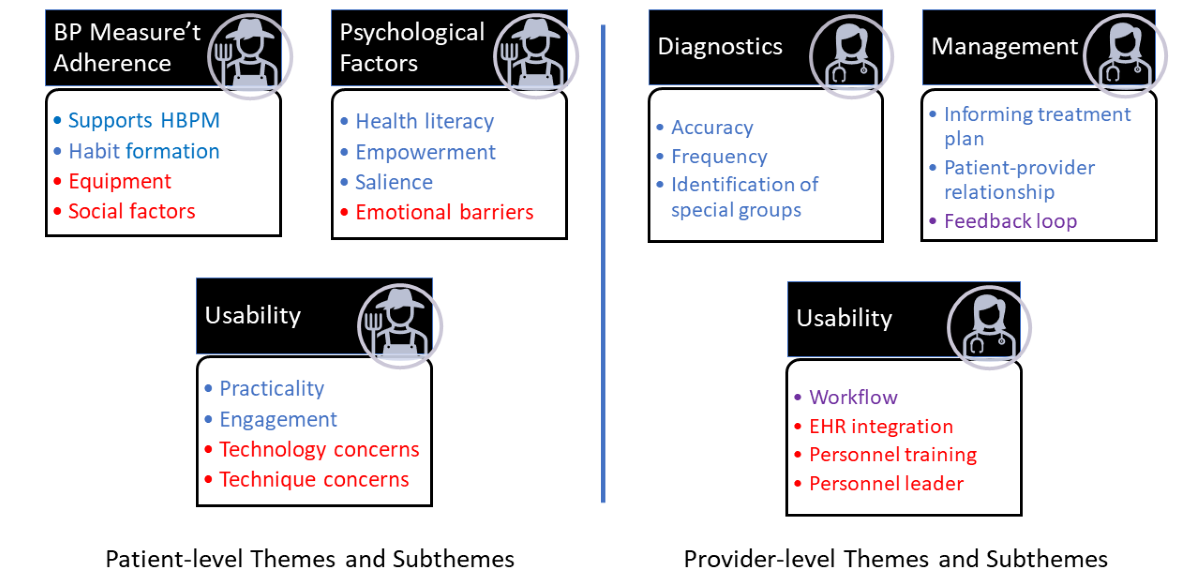
Patient- and provider-level themes and subthemes for codes generated by inductive transcript analysis of primary care provider interviews. Implementation facilitators are indicated by blue font, barriers by red font, and purple represents subthemes that may act as both barriers and facilitators. BP: blood pressure; EHR: electronic health record; HBPM: Home Blood Pressure Monitoring; Measure’t: measurement.

After this consensus was reached, the codes were analyzed to form an illustration or “mind map” of the interrelationships of the underlying thoughts and ideas related to each code (Figure 1). This was done without any a priori model or framework (ie, without reference to CFIR). The activity required iterative discussions between investigators to agree upon themes and subthemes based upon similarities between codes to develop a patterned response from the data set [30]. Themes and subthemes were refined to ensure that they were internally consistent and distinct from one another. As a second step, codes and themes were then reviewed and aligned to the CFIR framework. Representative examples were then selected from interview transcripts to denote findings. The data sets generated during this study are available from the corresponding author on reasonable request.

### Ethical Considerations

This study was approved by the University of Pittsburgh investigational review board (STUDY19050151). Participants received no payment, and all provided signed, informed consent. Personal identifying information is retained in a password protected file and paper documents in a locked cabinet.

## Results

### Overview

A total of 17 providers agreed to participate and were interviewed for an average of 31 (range 16 to 56) minutes. Participants included 11 physicians and 6 nurses or medical assistants from urban and suburban practices in the Pittsburgh metropolitan area. All 17 interviews were transcribed and included in the analysis. Inductive analysis was applied to generate a mind map of themes and subthemes without any a priori organization or model. This thematic analysis is displayed in Figure 1. Next, unmapped codes were aligned with CFIR. CFIR is organized in 5 domains, each of which contains multiple defined constructs. All 5 CFIR domains were represented in our results below and within each heading we list the subset of constructs under which those findings align.

### Innovation Domain: Relative Advantage, Complexity, and Design

Providers perceived a relative advantage for MyBP, reporting that implementing MyBP would offer patients broad support in adhering to systematic HBPM. Further, 1 provider (senior male physician, medium size urban practice) mentioned adherence with HBPM has “… gotta be over 90% I would assume…” compared to the “…50-60% compliance rating on patients without MyBP.” MyBP was envisioned as a tool providers could easily imagine using to broadly support BP measurement adherence. Other providers felt that MyBP would assist their patients in setting up a routine and then support adherence. Habit formation was frequently mentioned as another benefit of MyBP, helping patients incorporate systematic HBPM into their daily activities. Using such a set schedule for HBPM gives the patient “… a pretty clear standardized process for the patients to see …” in routinizing their measurement of BP. A senior male physician (small size rural practice) believed that creating a “stable schedule and also sending them messages to remind them to do it is even better… Because you know, you would have a certain amount that would say ‘Oh, I was going to do it but I forgot.’” Another provider (senior male physician, large academic practice) suggested that the utility of the MyBP program structure was to “[remind] the patient this is something important … help[ing] with compliance.” Providers thought that MyBP would help patient motivation.

By providing between-visit care, MyBP was felt to increase the daily salience of BP control to patients. This program ensured that they would continue to measure and remain aware of their BP despite long gaps between primary care appointments. A senior female physician (large academic practice) noted: “It gets them more involved. It helps them see what the point is ... it just makes their health stay on their radar a little bit more. I said ‘out of sight out of mind’, it’s kind of on them too as soon as they walk out of my office they don’t see me again often for 3 or 4 months.” By taking continuous measurements, patients may take more notice of and further understand the importance of these values. An early career female physician (large academic practice) noted, that MyBP “gives the patient something they can see, like a running tally …” and that “having the averages come back is helpful because they can see that and they know what’s happening with their BP.” Allowing patients to measure their BP in a convenient manner, within the comfort of their home was also considered a relative advantage: “[MyBP offers] convenience for the patients to take a BP reading whenever it’s reasonable for them. Not having to drive somewhere else to get it done” (female nurse, small suburban practice). MyBP was viewed as a practical method of obtaining the necessary BP data. A female nurse (large academic practice) stated that, “… it keeps the person on top of their numbers so that they know what they need to do, if they need to adjust their treatment or get treatment.” It was emphasized that using patients’ desire for more immediate and concrete feedback was an added benefit of MyBP: “…people like the idea of having interaction … they want to do good … You want to get the A, you want reinforcement…” (midcareer female physician, medium size urban practice).

Many thought these measurements obtained from home were a more reliable source of information and were better able to support management decisions. MyBP was noted by providers to enhance their diagnostic precision while managing hypertension. It helped that “the patients … were compliant and even though [their BP readings] were high here in the office, they got good readings at home” (midcareer female physician, medium size urban practice). Further, 1 provider remarked that MyBP helped to confirm a patient’s response to medication that was doubted after elevated in-office readings: “the numbers I was getting were actually better than um, I think the patients had been doing during clinic visits. And so, it seems like the blood pressures came down” (senior male physician, large academic practice). This helped providers to clarify the management of patients with previously inappropriately categorized or overtreated hypertension, including those with white coat hypertension. A senior male physician (medium size urban practice) found that MyBP influenced their treatment plans to manage hypertension: it “did a good job…and it did influence my treatment [plan].” Another senior male physician (medium size suburban practice) offered that MyBP “often times it makes the difference between starting somebody on medication or adjusting medication” due to the availability of program-generated home BP data. A senior male physician (medium size urban practice) observed that MyBP aided in some decision-making “in terms of who to treat or who to treat more aggressively, who not to treat more aggressively,” and another senior male physician (medium size suburban practice) noted the program “would be very very beneficial … [in] identifying overtreatment as well.” These promising insights indicated that providers felt comfortable modifying drug therapy with the help of the program.

In terms of complexity from the provider perspective, when discussing MyBP’s features, a female nurse (large academic practice) noted that [MyBP] “is a pretty simple format that … [is] not cutting into my busy schedule” and “I think that that would … enhance maybe compliance with this.” However, complexity was also seen as a barrier from the patient perspective. Overall, it was felt that MyBP was easy for patients to use. The same nurse noted that, “I think it’s great because I think it is simple… And I think folks need simple.” An early career female physician (small suburban practice) added, “I’m someone who isn’t super tech savvy, but I think I could easily do this … it seems pretty simply designed.” However, there was concern that patients struggling with other self-management behaviors may have difficulty using MyBP. A female nurse (large academic practice) observed, “you have to have an engaged patient who wants to take care of themselves and wants to learn more in order to use the tool that’s there and if they don’t see the benefit, they’re not gonna use it.”

### Outer Setting Domain: External Pressure, Societal Pressure, and Financing

There were several outer setting barriers that providers noted at the societal level. An early career female physician (small suburban practice) stated, “we already have some barriers to controlling their blood pressure, and I don’t know if this would necessarily address… concerns about being able to afford medications, not going for refills, those kinds of things.” A senior female physician (large academic practice) used the story of a particular patient to model a larger point, explaining that she is at the “crux of one of our greatest challenges in our healthcare system … the source of the tremendous difficulty isn’t medical; it’s her life, it’s social…” Finally, she expressed skepticism: “I don’t know if at the level of MyBP we can address the hot spotter population.”

Likewise, insurance policies were considered to be a potential outer setting barrier to the implementation of MyBP. Access to a personal BP monitor was a repeatedly mentioned barrier: “not having a blood pressure cuff and acquiring a blood pressure cuff is a barrier … even if we jump through hoops and so forth, very few people get a blood pressure cuff through their insurance” (female nurse, large academic practice). This provider continued to explain that often those most impacted by socioeconomic barriers are the ones most in need of access to tools such as the BP device, further highlighting the interplay between access to resources and differences in health outcomes.

### Individuals’ Roles Domain: Need, Capability, Opportunity, and Motivation

Providers perceived that patients have low motivation or capability. Further, 1 midcareer female physician (medium size urban practice) said, “often they [patients] don’t get the cuff when you ask them to, or they don’t know where to get the cuff at.” Although some providers mentioned patient health literacy as a potential barrier to MyBP (capability), they reported that the educational tools provided by the program could address this barrier. MyBP’s educational videos assisted with learning “… about how to check proper pressures, and how to do … the proper technique, and uh just explaining the meanings behind those numbers …” This senior male physician (medium size suburban practice) continued, “in the 27 years I’ve been doing this … I’ve realized the more that you explain to people and give them information to use the less likely they are to have problems, and … when they do, they understand … what the issue is.”

Providers perceived that patients were motivated but that education would be needed to ensure that this did not result in anxiety: “When they’re getting a bunch of numbers and they’re not really understanding what those numbers mean, I think a potential is there for people to get um scared or excited or something…” (female nurse, large academic practice). Therefore, ensuring that patients have the context to interpret these values and manage adverse emotional responses was of great importance to the success of MyBP. Another provider noted a similar barrier when patients become overly fixated on knowing their BP at any given point in time: “I mean some are really—some are really diligent, I mean some, some- some people I think kinda overdo it. And they take their blood pressure more than I think they need to” (senior male physician, large academic practice). In summation, providers foresaw circumstances where MyBP could cause stress that may deter patients from a constructive HBPM experience.

Other interviewees noted concern about the technology’s capability for some patients. While they viewed texting as convenient, an early career female physician (small suburban practice) was still unconvinced “… about the folks that aren’t tech-savvy enough.” This same provider felt the interface “would be difficult for them to manage,” and a senior male physician (small size rural practice) went on to say “Well, there will be a group that doesn’t want to fool around with that. But, um, I mean I still have mine that come in with written pressures all the time...” An adjacent concern included the belief that the use of texting within the MyBP program could lead to input errors. While MyBP was designed to avoid reliance of automated data collection (eg, with Bluetooth), several providers viewed the step of inputting the BP reading or interfacing with MyBP via SMS text messaging as a barrier.

Patient measurement technique error was also a concern as an inability to verify proper technique for each BP measurement. A female nurse (small suburban practice) voiced that “I’d wonder about accuracy overall. I’m sure there’s still room for patient error.” A senior female physician (large academic practice) felt that using the tool raised more questions: “[there was] more that I would’ve wanted to know when I got the reports … Was she seated at rest for five minutes? Was she checking in her left arm? Was she checking her blood pressure properly?” While providers felt MyBP would be beneficial in increasing the amount of BP data they could use to inform their treatment plan, the lack of contextual information could lower confidence in the BP data.

They did report that MyBP increased patient opportunity to check BP and increase accuracy. “It’s more accurate BP readings than what we get in the hospital. More accurate and more frequent. [*pause*] That they get to record their BPs more, than just once when they come to the hospital…” (female medical assistant, large academic practice).

### Inner Setting Domain: Informational Technology Infrastructure, Work Infrastructure, Relational Concerns, Communications, Structural Characteristics, Recipient-Centeredness, Deliverer-Centeredness, Tension for Change, Compatibility, and Available Resources

MyBP was adaptable, which addressed inner setting barriers including time. Providers also found that MyBP helped to elucidate a patient’s “true” BP: “patients are able to do it on their time … in a relaxed setting. You get a better picture of their blood pressure. It’s hard for us to do that whenever they only come in at a specific time” (female nurse, small suburban practice). They noted confounding variables with in-office measurements, such as when the patient is “so out of breath of just came up 2 flights of steps or something like that” (female nurse, large academic practice).

In addition, providers felt that MyBP enhanced their relationship with their patients, improving their ability to develop rapport. This was noted through a senior male physician’s (medium size urban practice) observation that patients “were asking me whether I had seen the reports…and they wanted to know what I thought” with another provider (early career female physician, large academic practice) observing their patients “were pretty excited to show what they’ve been doing with their BPs.” The program reports gave patients an opportunity to engage with their providers and interact beyond typical care interactions. The program provided a platform for patients to not only be held accountable themselves for their HBPM measurements, but also to keep the clinical team accountable, through asking if they had received faxes and initiating more dialogue about their BP.

Patient-provider feedback loops were frequently discussed, not only in terms of how they were improved by the program but also how some issues persisted despite it. A female nurse (large academic practice) felt that they were “getting results in a timely fashion,” which is important because “you can’t fix what you don’t see.” Another female nurse (large academic practice) mentioned that the program allowed for “an opportunity for earlier intervention, for medication changes as opposed to waiting, again, another 3 or 6 months.” They felt that the between-visit care was improved by virtue of additional data points to overcome clinical and diagnostic inertia. Treatment teams appreciated the advantage of the communication channels offered by the program, especially in its lowering of the required investment of patient time and effort to receive at least some BP information and guide therapy.

There were several providers that noted positive tension for change, such as a senior male physician (small size suburban practice) saying “I think, it will just kind of make more structure to something that I’m doing … Anything to support that or give structure to something, I feel is important … could be helpful.” Another senior male physician (small size rural practice) reflected that the data from the program “would be just nice and more formalized.” They would already have “the data aggregated so that I wouldn’t have to be calculating averages myself” (early career female physician, small suburban practice) as opposed to the current status quo, in which data collection is more piecemeal and nonaggregated, taking “additional time … away from the nurses” (female nurse, small suburban practice). Providers felt that the ability of the program to quickly aggregate and make useful conclusions about BP data offered a significant benefit from a time and effort standpoint.

However, interviewees did believe that uptake of the fax reports and updating clinical practice patterns would take some effort. Speaking to concerns about compatibility with the current clinic flow, a senior male physician (large academic practice) pointed out, “the clinic staff already has a million things to do and so the process would have to be streamlined somehow,” and that “you kind of have to … modify that process to what would work in an actual clinic.” Providers felt a significant push would be needed to overcome the inertia within clinical practices and begin to change workflow. Regarding fax reports an early career female physician, small suburban practice, said, “I do get worried about getting information by fax … I’m [away from the clinic] for 2 weeks and … I [c]ould miss something that I would want to see.” While a senior male physician (medium size rural practice) commented “And now we’re buried in paper again.” Interviewed providers believed that direct electronic health record (EHR) integration would aid in implementation of the platform.

Barriers to clinical implementation of MyBP also extended to personnel issues such as the need to expand office staff. A senior male physician (medium size urban practice) reflected that “if we had the staff then I would say we were we we [*sic*] could implement it but we need some additional sta—team members to help us do it” and that hiring new staff presents a “tremendous workload.” A female nurse (small suburban practice) felt the “training for staff” was the most important issue facing her practice, as it was difficult for her to make “sure that staff were also properly aware of application of the cuff and all of that …It just goes back to staff education.”

### Implementation Process Domain: Teaming, Planning, Engaging Innovation Deliverers, and Adapting

It was believed a local “champion” for MyBP would be beneficial in establishing enthusiasm and having the program started at each site. A female nurse (small suburban practice) liked the idea of having a “point person for it so somebody is keeping the list [of enrolled patients] together…” or (senior female physician, large academic practice) if a “MyBP person was housed on site … and summed up the BP reports inter-visit…” Having dedicated MyBP personnel to help with the normal workflow of the program and assist with troubleshooting seemed overall to be beneficial.

There were also safety concerns regarding implementation, speaking to the need for a process for data return. Instead of the monthly report cycle, a midcareer female physician (large academic practice) expressed that they “would have liked more feedback sooner… Sometimes I didn’t get it for a while and making a change took time.” This thinking also applied to more urgent patient scenarios, in which an early career female physician (small suburban practice) expressed that they would feel concerned “…if I miss data that tells me someone has had very high blood pressures, and no one has checked in to see about symptoms or no one has made a move to try to control their blood pressure better.” Although providers saw the between-visit BP data as an advantage compared to standard follow-up, they had persistent concerns that symptoms or extreme BP values would go unnoticed by office staff for several weeks.

## Discussion

### Principal Findings

This study qualitatively assessed providers’ perceptions of the implementation of MyBP, a text-message based program designed to engage patients in ongoing hypertension self-management focused on systematic and continuous HBPM. Given the near-universal competency in using text messaging including among older adults, mobile health (mHealth) using automated SMS text messaging may be effective for population health interventions [32] and specifically in disadvantaged patient populations [27,33]. This report complements patient stakeholder evaluation of MyBP based upon 40 interviews [26]. Here, the transcripts of semistructured interviews to physicians, nurses, and medical assistants were analyzed using pattern identification of concepts to generate de novo codes and themes classified as pertaining to either the patient experience or the practice or provider experience. We then returned to the initial codes and themes to map the findings onto the 5 CFIR domains [29].

Providers felt that MyBP would offer strong foundational support in a patients’ efforts to systematically monitor their BP at home. They believed SMS text messaging would facilitate sustainable health-behavior habits without infringing into patients’ schedules. This is enhanced by patients' access to video-based educational materials and improved health literacy. As such, providers believed the program helped patients feel more in control of their BP, and in turn view their high BP as more manageable. Providers also stated that the program is practical for patients to use as it connects patients to their health care from the comfort of their homes. This decreased the need to spend time or money on travel to a clinic for BP checks, further supporting a patient’s engagement with their BP management. These themes relate to the CFIR Innovation Domain constructs of relative advantage and design. This evidence corroborates patients’ reports that MyBP may strengthen their self-efficacy [26], and our quantitative evidence of continued engagement with systematic HBPM aided by the program [27].

Nonetheless, providers saw barriers to implementation. One of the most voiced concerns was patients’ difficulties securing personal BP devices. As insurance generally does not provide coverage for BP cuffs, low-income patients are particularly affected. There was also concern that the attention given to HBPM could cause some patients to become fixated on their BP readings. Providers worried that such hypervigilance would become a source of distress. Notably, the MyBP program design discourages fixation by not accepting extra readings or readings outside of the self-selected time windows.

Regarding the basic design of MyBP, providers worried that deploying messaging through SMS text messaging would impede use by patients who do not wish to or are unable to use this technology. Patient self-submitted measurements via SMS text messaging may contain errors if done incorrectly. Alternatives include automated data uploading using a Bluetooth enabled monitor paired to the patient’s smartphone and newer cuffless, wearable devices. These approaches are limited by the digital divide and are likely to exacerbate health disparities. Furthermore, providers had concerns about the HBPM measurement technique. As patients would be taking their BP unsupervised, providers wondered if lack of oversight could result in unreliable BP readings.

From the providers’ perspective, their experience with MyBP was most notable for a perceived improvement in their ability to diagnose and manage hypertension. The increased number of measurements and more realistic setting in which the measurements were captured provides valuable data to guide intervisit care, as evidenced by providers’ repeated mention of the ease of navigation of the BP reports organized as successive 2-week averages. They expressed that their current practice team lacked a method for organizing large swaths of BP data into a digestible format. The program was also felt to help identify when patients were being over- or under-treated for hypertension, especially in the case of white-coat hypertension, all while improving provider confidence to overcome therapeutic inertia [34]. Providers also saw that patients involved in the program were likely to discuss their BP and use the application interface as a jumping off point for more health-related dialogue, taking more initiative in their hypertension management. In this sense, the program may increase accountability between provider and patient in a bidirectional manner.

Providers expressed concern about implementing the MyBP program within existing clinical workflows. They thought that the BP fax reports may prove cumbersome to sort through given their large patient volume and ongoing transition to paperless records, hindering the quick review of BP data. Often discussion of the fax reports led to an appeal for the integration of BP data directly into the EHR, though there was some skepticism regarding interoperability. Providers varied in their suggestions about where such HBPM data should be located within the EHR (ie, vitals, outside documents, or reports).

A desire for more dedicated training was frequently voiced. The goal would be to improve staff awareness and management of the program, as well as designation of a dedicated team member as the program “champion.” These concerns were exacerbated by the worry that with the reports generated monthly, there could be dangerously high or low BP values that were not addressed expeditiously. This may require close monitoring and scrutiny through additions to staff workflow. Collectively, the above themes constitute barriers to the implementation spanning CFIR Innovation Design and Cost, Inner Setting Work Infrastructure and Communications, and Implementation Process Teaming and Adapting.

Future iterations of the program should thus focus on these barriers of workflow integration and staff training, possibly including a local champion. Program modification to enable automated transfer of BP data into the EHR requires improved interoperability, although all values are already digitized and contain 2-week BP averages. These BP report summaries could be generated automatically in plain text and routed to that section of the EHR dictated by program design or user preference. Notably, incoming actionable health data can impact medicolegal liability. The barriers identified highlight that despite numerous advantages of the program in improving the diagnosis and management of hypertension via utilitarian patient interface, there is still a significant need for implementation supports and related resources. On the other hand, the application’s operation is more automated, efficient, and scalable than most other programs that support patients in HBPM [10].

The report contributes to an otherwise underdeveloped literature on implementation of programs for systematic HBPM. Prior qualitative research indicates many positive aspects of mHealth hypertension programs leading to improved self-management [35], whereas without a well-designed tool to assist with systematic home BP measurement patients often fail to comply with collection and reporting of HBPM data [36]. Even the best resourced and tested mHealth intervention for hypertension management was rated by providers as more challenging to implement than paper-based BP reports from patients [37].

Study limitations include the relatively small number of providers interviewed. Furthermore, some interviewed providers had had no direct experience with MyBP, though they did have patients performing HBPM. Further, most of those interviewed worked within the same health care network, such that their opinions may have limited generalizability. Stakeholder input from providers in diverse communities and clinical settings would generate additional stakeholder insight.

### Conclusions

Uncontrolled hypertension is a serious and highly prevalent condition for which new approaches are needed. Primary care providers felt that a program such as MyBP can support and improve patient engagement with HBPM and engagement with self-managing their hypertension, while concurrently gathering and organizing actionable data to guide prescribed pharmacotherapy. Notwithstanding this, providers also identified clinical and patient-centric barriers to implementation of the program in office practice settings. The themes of increased data quality and support for healthy habit formation in patients were lauded. Providers collectively described the need for additional supports and resources as well as adaptations to the program itself. These included staff training and workflow adjustments, better reporting flexibility and EHR interface, and parallel resources to cover the out-of-pocket cost of BP monitors. Given the widespread challenge of uncontrolled hypertension further health services research should advance the design and deployment of technology-leveraged programs supporting systematic HBPM and self-management support.

## Appendix

Multimedia Appendix 1 Supplementary materials.

## Abbreviations

BP: blood pressure
CFIR: Consolidated Framework for Implementation Research
EHR: electronic health record
HBPM: home BP monitoring
mHealth: mobile health
