# Supplementary material for: Automated Messaging Program to Facilitate Systematic Home Blood Pressure Monitoring: Qualitative Analysis of Provider Interviews
Source: JMIR Cardio. 2023 Dec 4;7:e51316. doi: 10.2196/51316 (PMC10728784; doi:10.2196/51316)
Supplement: Multimedia Appendix 1 [file cardio_v7i1e51316_app1.docx]

Provider and Staff Interview

**Impression & Beliefs about Home BP monitoring/Tension for Change**

1. What are your thoughts about home BP monitoring? (If not answered: What do you find useful about it?) What challenges have you encountered when patients are asked to measure their own BP?
2. Do you see a need for more or better home BP monitoring?

**External Policies & Incentives**

1. Are there any policies or performance measures (UPMC, state, or national) that influence your use of home BP monitoring? How do performance evaluations or incentive pay influence your use of home BP monitoring if at all?

**Patient Needs & Resources**

1. How well do you think that home BP monitoring meets the needs of your patients? (Tie question in to answer from #1 when applicable)
2. How well do you think that MyBP supports home BP monitoring for your patients?
   - In what ways? For example, does it improve patient activation or empowerment? … improve provider-patient alliance or communication? … reduce travel time and expense?
3. In context of your answer about the challenges you encountered when asking patients to measure their own BP (#1), what do you think the underlying barriers are behind that low compliance?
4. How well do you think MyBP supports home BP monitoring for your needs specifically? (If not mentioned: What did you think of the faxed reports?)
5. Have you heard stories about the experiences of participants using MyBP? Can you describe a specific story?

**Relative Advantage**

1. Besides home BP monitoring, providers can use office or 24hr ambulatory BP; what do you see as the pros and cons of each in your practice? (If not mentioned: What about reliability?)
2. Now considering home BP monitoring *with* MyBP, does that change the pros/cons you just mentioned? (When applicable: In what way?)
   - What advantages does MyBP have for providers/staff? (compared to existing programs) What is the best thing about MyBP?
   - What disadvantages does MyBP have compared to existing programs?

**Adaptability**

1. Ignoring MyBP for the moment, what kinds of changes or alterations do you think you will need to help increase or improve home BP monitoring in your practice? (Aside from free cuffs if mentioned earlier ->) Do you think you will be able to make these changes? Why or why not?
2. Now, considering how MyBP is designed, including the program and instructions, what would make it work better? Probe: Are there components that should be changed? (If not mentioned: What do you think of the quality of the texting program and instructions?)

**Design Quality & Packaging**

1. I mentioned some of MyBP’s supporting materials earlier, such as the educational videos and printed instructions, what additional supports from MyBP, would be helpful for implementing home BP monitoring in your setting?

**Available Resources**

1. Considering your current use of home blood pressure monitoring, how do you tackle factors such as patient education regarding hypertension and proper HBPM technique? Do you think MyBP addresses those? (e.g. through repetition or reinforcement via texts and videos)

Are there any resources that you have received so far or are hoping to receive to aid your use of home BP monitoring with your patients? (If applicable: How easy was it to obtain those resources?) Other than training or possibly a staff member devoted to MyBP, do you think you would need additional resources to implement MyBP in your setting?

(If not mentioned, what are your thoughts on: on-site staff training, a MyBP champion among their staff, financial incentive/reimbursement, free BP cuffs, a HELP phone number, a dashboard or some other report for all their enrolled patients, rather than single patient reports?)

**Structural Characteristics**

1. What kinds of infrastructure changes will be needed to better accommodate home BP monitoring? (If not mentioned: Do you think there is a need for more structure or in your current use of home BP monitoring?) Would you need to make infrastructure changes to include MyBP in your home BP monitoring program? If so what are these? Changes in formal policies? Changes in information systems or electronic records systems? Other?

**Self-efficacy**

1. How confident are you that you will be able to help your patients implement home BP monitoring? What gives you that level of confidence (or lack of confidence)? How confident are you in your ability to use MyBP in this process?

**Individual Stage of Change**

1. How prepared is your office to implement MyBP if it were to become available next month? What changes or what steps you would take if it were to become available next month?

**Consolidated Framework for Implementation Research (CFIR) Domains and Constructs***

The constructs included in the interview are indicated with underlining.

1. **Intervention Characteristics**
2. Intervention Source
3. Evidence Strength & Quality
4. Relative Advantage
5. Adaptability
6. Trialability
7. Complexity
8. Design Quality & Packaging
9. Cost
10. **Outer Setting**
11. Patient Needs & Resources
12. Cosmopolitanism
13. Peer Pressure
14. External Policies & Incentives
15. **Inner Setting**
16. Structural Characteristics
17. Networks & Communications
18. Culture
19. Implementation Climate
20. Tension for Change
21. Compatibility
22. Relative Priority
23. Organizational Incentives & Rewards
24. Goals & Feedback
25. Learning Climate
26. Readiness for Implementation
27. Leadership Engagement
28. Available Resources
29. Access to Knowledge & Information
30. **Characteristics of Individuals**
31. Knowledge & Beliefs about the Intervention
32. Self-efficacy
33. Individual Stage of Change
34. **Process**
35. Planning
36. Engaging
37. Executing
38. Reflecting & Evaluating

(*) The 2009 version was used since the interview was created prior to release of the 2022 version.
